# Skeletal Muscle Depletion and Major Postoperative Complications in Locally-Advanced Head and Neck Cancer: A Comparison between Ultrasound of Rectus Femoris Muscle and Neck Cross-Sectional Imaging

**DOI:** 10.3390/cancers14020347

**Published:** 2022-01-11

**Authors:** Andrea Galli, Michele Colombo, Carmine Prizio, Giulia Carrara, Francesca Lira Luce, Pier Luigi Paesano, Giovanna Della Vecchia, Leone Giordano, Stefano Bondi, Michele Tulli, Davide Di Santo, Aurora Mirabile, Francesco De Cobelli, Mario Bussi

**Affiliations:** 1 Department of Otorhinolaryngology, San Raffaele Scientific Institute, Via Olgettina 60, 20132 Milan, Italy; c.prizio@studenti.unisr.it (C.P.); liraluce.francesca@hsr.it (F.L.L.); giordano.leone@hsr.it (L.G.); bondi.stefano@hsr.it (S.B.); tulli.michele@hsr.it (M.T.); bussi.mario@hsr.it (M.B.); 2 Department of Radiology and Experimental Imaging Center, San Raffaele Scientific Institute, Via Olgettina 60, 20132 Milan, Italy; colombo.michele@hsr.it (M.C.); paesano.pierluigi@hsr.it (P.L.P.); dellavecchia.giovanna@hsr.it (G.D.V.); decobelli.francesco@hsr.it (F.D.C.); 3 Department of General Surgery, Ospedale Fatebenefratelli e Oftalmico, Piazzale Principessa Clotilde 3, 20121 Milan, Italy; giulia.carrara@asst-fbf-sacco.it; 4 Department of Otorhinolaryngology, Humanitas Research Hospital, Via Manzoni 56, 20089 Rozzano, Italy; davide.disanto@humanitas.it; 5 Department of Medical Oncology, San Raffaele Scientific Institute, Via Olgettina 60, 20132 Milan, Italy; mirabile.aurora@hsr.it

**Keywords:** sarcopenia, ultrasound, rectus femoris, cross-sectional imaging, head and neck cancer, major complications

## Abstract

**Simple Summary:**

Skeletal muscle mass (SMM) depletion is gaining popularity as independent predictor of postoperative complications in many surgical scenarios, even in the field of head and neck oncology. In this study, we demonstrate the value of ultrasound scans of the rectus femoris muscle together with the neck CT/MRI at C3 level in terms of estimation of SMM (through muscle cross sectional area), the identification of sarcopenic patients and as a predictor of major surgical morbidity in a cohort of locally-advanced head and neck cancer patients submitted to surgical treatment. This provides important tools for the on-going re-assessment of patients with regards to any pre-habilitation strategy aimed at reducing postoperative complications.

**Abstract:**

Skeletal muscle mass (SMM) depletion has been validated in many surgical fields as independent predictor of complications through cross-sectional imaging. We evaluated SMM depletion in a stage III-IV head and neck cancer cohort, comparing the accuracy of CT/MRI at C3 level with ultrasound (US) of rectus femoris muscle (RF) in terms of prediction of major complications. Patients submitted to surgery were recruited from 2016 to 2021. SMM was estimated on CT/MRI by calculating the sum of the cross-sectional area (CSA) of the sternocleidomastoid and paravertebral muscles at C3 level and its height-indexed value (cervical skeletal muscle index, CSMI) and on US by computing the CSA of RF. Specific thresholds were defined for both US and CT/MRI according to ROC curve in terms of best prediction of 30-day major complications to detect sarcopenic subjects (40–53%). Sixty-five patients completed the study. At univariate analysis, major complications were associated to lower RF CSA, lower CSA at C3 level and lower CSMI, together with previous radiotherapy, higher ASA score and higher modified frailty index (mFI). At multivariate analysis RF CSA (OR 7.07, *p* = 0.004), CSA at C3 level (OR 6.74, *p* = 0.005) and CSMI (OR 4.02, *p* = 0.025) were confirmed as independent predictors in three different models including radiotherapy, ASA score and mFI. This analysis proved the value of SMM depletion as predictor of major complications in a head and neck cancer cohort, either defined on cross-sectional imaging at C3 or on US of RF.

## 1. Introduction

Head and neck squamous cell carcinoma (HNSCC) is diagnosed in over 550,000 patients per year globally and is responsible for over 380,000 deaths [1]. Though 60% of patients are diagnosed at advanced stages with a consequently high risk of local recurrence and distant metastasis, patient-specific factors, such as age and comorbidities, also significantly impact the overall prognosis of these diseases [2,3]. These indeed affect the capability of patients to undergo heavy multimodal therapies, which are the gold standard of treatment for such extended disorders, jeopardizing their chances to be healed [4]. Moreover, approximately 35% to 60% of all patients with HNSCC are malnourished at the time of their diagnosis because of cancer itself and its treatment modalities [5,6]. Friedlander et al. found that the degree of malnutrition correlates with the tumor burden and affects outcome; furthermore, the immune suppression observed in malnourished patients is associated with unimpeded tumor growth [7,8]. Malnourished and cachectic patients are also frequently unable to tolerate antineoplastic therapies, resulting in treatment delay and higher costs [9].

More specifically than malnutrition, sarcopenia reflects the poor general conditions frequently experienced by patients affected by locally-advanced cancer, representing a decline in skeletal muscle mass (SMM) concomitant with decreased strength and/or function [10]. SMM depletion, as surrogate marker of sarcopenia, has been widely validated as risk factor for treatment-related major complications and mortality in many oncological fields, especially general surgery. In this specific scenario, cross-sectional imaging techniques, such as computed tomography (CT) and magnetic resonance imaging (MRI), have been the elective methods to compute SMM at the level of the third lumbar vertebra (L3) and stratify patients accordingly [11,12,13,14,15]. This L3-based technique has traditionally limited its application to the head and neck field, though HNSCC patients are between those at the highest-risk for the development of sarcopenia, especially at locally-advanced stages [9]. Recently, Swartz et al. demonstrated the feasibility of SMM estimation using head and neck CT scan at the level of the third cervical vertebra (C3) as an alternative to the L3-based technique routinely performed on abdomen CT scan [16]. Nevertheless, evidence on this topic is still limited.

Our group previously assessed the potential role of the rectus femoris muscle ultrasound (US) in terms of muscle cross-sectional area (CSA) as preoperative surrogate marker of sarcopenia within a locally-advanced head and neck cancer (LA-HNSCC) cohort of patients submitted to surgical treatment in terms of prediction of postoperative complications and poor prognosis [17]. The aim of the present study was to confirm the value of rectus femoris US as a prognosticator in an expanded cohort of patients and to compare it to the commonly employed assessment based upon neck cross-sectional imaging (CT, MRI) for the prediction of 30-day major postoperative complications.

## 2. Materials and Methods

### 2.1. Selection Criteria and Data Collection

**Figure 1:**
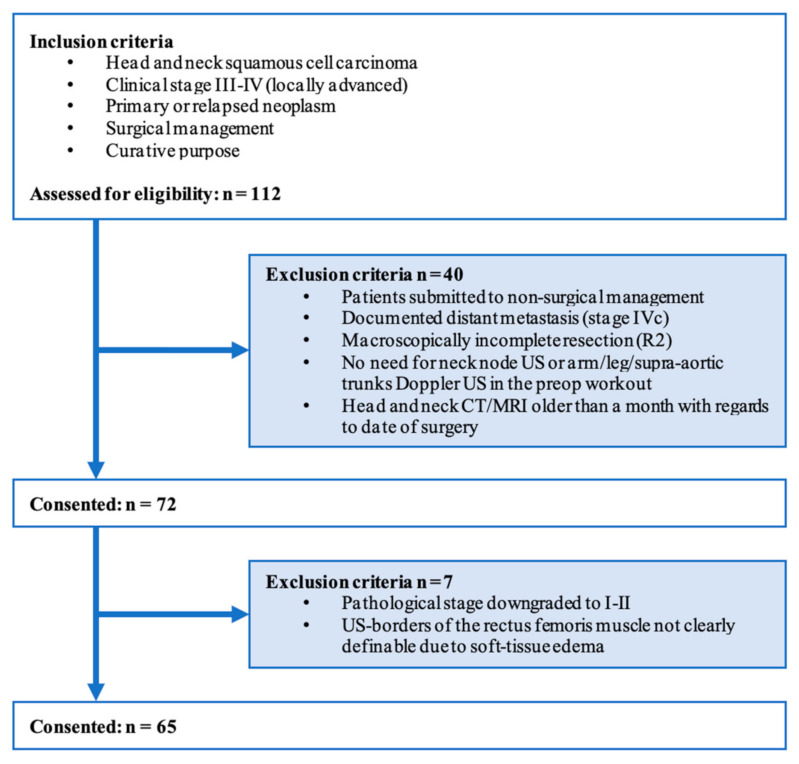
Study flow diagram.

We evaluated our series of 112 consecutive patients affected with clinical stage III-IV (AJCC/UICC TNM Classification of Malignant Tumors, eighth edition, 2017) [18] HNSCC submitted to surgical treatment with curative purpose between November 2016 and January 2021 at the ENT department of San Raffaele Scientific Institute (Milan, Italy). The study was retrospective, observational, case-control in design. All patients were discussed in the local multidisciplinary tumor board (composed of ENT surgeons, medical oncologists, radiation oncologists, head and neck radiologists, nuclear medicine physicians, pathologists) before and after surgery for proper clinical indications. Patients submitted to nonsurgical treatment, with documented distant metastasis or operated with macroscopically incomplete resection (R2 margins) were ruled out. We also excluded patients whose preoperative workup did not require neck node US (done with regional staging purpose) or arm/leg/supra-aortic trunks Doppler US (done with reconstructive purpose, for free flap harvesting), since the US of rectus femoris muscle was performed during those planned exams. Subjects whose clinical stage (cTNM) was downgraded to pathological stage (pTNM) I–II because of intraoperative/permanent sections were likewise excluded. A study flow diagram is depicted in Figure 1.

All the patients were submitted to postoperative enteral nutrition with nasogastric tube (NGT), except for those subjected to extended parotidectomy who started oral nutrition on the first postoperative day. The nutritional program was defined according to the estimated total energy expenditure on the advice of a dedicated nutritionist. Tube weaning and shift to oral nutrition was performed after specific bedside swallowing evaluation. No parenteral intervention, immune-nutrition or oral supplements were provided.

All performed procedures and data management were in accordance with the ethical standards of the national research ethical committee and with the principles and guidelines stated in the 1964 Declaration of Helsinki and its later amendments. The present study protocol was evaluated by the IRB at the clinical research center where it has been conducted (protocol number: HeadNeckSurg, 181/INT/2021; approval date 15 December 2021). Informed consent was obtained from all the enrolled patients.

### 2.2. Ultrasound of Rectus Femoris

**Figure 2:**
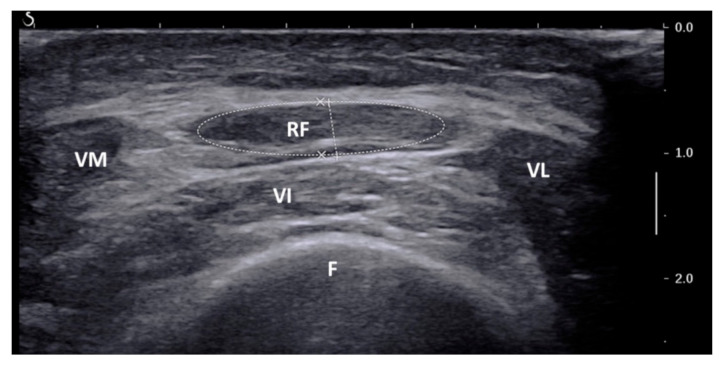
Ultrasound image of the rectus femoris muscle with definition of cross-sectional area (dashed line); RF: rectus femoris; VI: vastus intermedius; VL: vastus lateralis; VM: vastus medialis; F: femur.

US of the rectus femoris muscle was performed on the eve of surgery by a single, dedicated radiologist (M.C.), with B-mode ultrasonography (modular probe: 9–15 MHz), with the same standard protocol already published [17,19]. The patients were placed in supine position, with legs extended and muscles relaxed, and a point between middle and distal third of a line conducted between the anterior-superior iliac spine and the superior border of the patella of the dominant thigh was identified. The US probe was positioned perpendicularly along the superior aspect of the dominant thigh and a transverse image of the rectus femoris muscle was frozen (Figure 2).

This assessment was performed during planned preoperative neck node US (done with regional staging purpose in patients with uncertain cN status according to previous radiological studies) or arm/leg/supra-aortic trunks Doppler US (done with reconstructive purpose for free flap harvesting), so as not to add further procedures to the expected diagnostic workup. CSA was computed with planimetric technique (provided by US software) after manually tracing the inner echogenic line of rectus femoris muscle on the frozen image. Since normal muscle mass differs by sex, CSA values were corrected ×1 for male (reference group) and ×1.484 for female, according to Mueller et al. [19] (i.e., sex-adjusted CSA). The whole process added about 5 min to the expected procedures, without further biological risk for the patients.

### 2.3. CT/MRI at C3 Level

**Figure 3:**
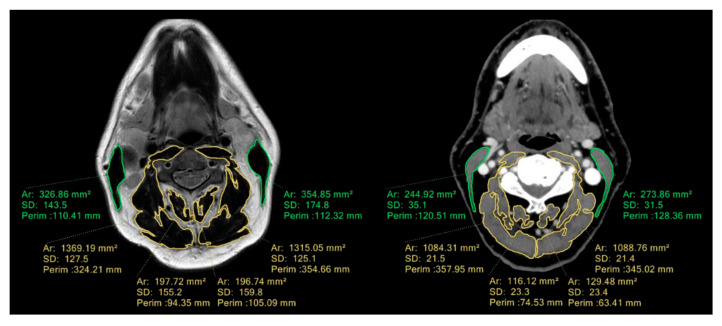
Segmentation of sternocleidomastoid (green) and paravertebral muscles (yellow) at C3 level using MRI (**left**) and CT (**right**) imaging in two different patients.

For SMM estimation, we used head and neck CT/MRI performed for loco-regional staging purposes within one month from the date of surgery. Regions of interest (ROIs) were drawn using the semi-automated software Philips IntelliSpace Portal 9.0 (Philips Healthcare, Best, The Netherlands). A predefined protocol for single slice selection and measurement of CSA of SMM was provided, as described by Swartz et al. [16], with C3 as reference point. Slice selection was performed by scrolling through the C3 vertebra in a caudad to cephalad direction and selecting the first slice to completely display the entire vertebral arc and the transverse and spinous processes, as shown in Figure 3.

For CT scan, the SMM area was identified as the pixel area within a radiodensity between −29 and +150 Hounsfield units (HU), which is recognized as a specific interval for skeletal muscle tissue [20]. Necessary corrections were performed manually by a single researcher (C.P.). After delineation, the CSA was automatically retrieved as the total sum of delineated pixels. For MRI, SMM area was manually segmented by a single researcher (C.P.), who personally delineated muscle tissue from the fatty area. An example of segmentation at C3 level is shown in Figure 3. CSA of the paravertebral muscles (PVM) and sternocleidomastoid (SCM) muscles were measured separately, as the SCM muscles could occasionally be invaded by lymph node metastases impairing CSA measurement; in such cases, the duplicated value of normal contralateral SCM was used. The whole process required 5 to 10 min for each patient. CT/MRI-defined cervical SMM was calculated as the sum of the delineated CSA of the PVM and SCM muscles. CT/MRI-defined cervical skeletal muscle index (CSMI) was calculated by correcting cervical SMM for squared height, as shown in the Equation (1):

Cervical SMI (cm^2^/m^2^) = CSA at C3/length (m^2^). (1)

### 2.4. Other Clinical Predictors

Aside from radiological variables, other clinical parameters were collected from patient interviews and preoperative blood tests: age, previous head and neck radiotherapy performed for the index tumor, BMI, smoking habit, history of diabetes mellitus, unplanned weight loss in the previous 3 months, hemoglobin, albumin, cholinesterase, creatine phosphokinase. No patient-based screening of nutritional risk was employed since most of our patients would have achieved overlapping high values because of the specific features of the enrolled disease. Surgical time was also recorded, as well as pN class, pTNM stage and eventual free flap reconstruction. Comorbidity assessment was performed according to the American Society of Anesthesiologists (ASA) score, the age-adjusted Charlson Comorbidity Index (CCI) and the modified Frailty Index (mFI).

### 2.5. Clinical Endpoint: 30-Day Major Postoperative Complications

The main variable of outcome was 30-day major postoperative complications, conditions that significantly affect overall hospital stay and costs. They were defined as those ≥3 according to the Clavien-Dindo classification (requiring surgical reoperation, ICU admission or leading to death) [21], all flap failures and all fistulas, either requiring surgical reintervention or prolonged conservative management for complete healing. Hospital stay was recorded as the time in days between the surgical procedure and the day of hospital discharge.

### 2.6. Statistics and Data Management

Association analyses between 30-day major postoperative complications and independent predictors were performed through crosstabs and Chi squared test or Fisher’s exact test, when appropriate, for categorical or dichotomized variables and through non-parametric Mann-Whitney *U* test for continuous variables. Association analysis between hospital stay and 30-day major postoperative complications was performed through non-parametric Mann-Whitney *U* test.

As stated, US-defined rectus femoris CSA, CT/MRI-defined cervical SMM and CSMI were used as surrogate markers of sarcopenia to stratify patients. The proper CSA thresholds for definition of low SMM (i.e., sarcopenic subset) were determined according to receiver-operating characteristic (ROC) curve in terms of best prediction of 30-day major postoperative complications. Traditional published cut-offs were used to dichotomize continuous variables, when feasible. ROC curves were used to define discrete thresholds in terms of best prediction of 30-day postoperative complications for other continuous variables. Multivariate analysis with multiple logistic regressions was performed with the main variables of outcome as endpoint. SPSS statistical package (IBM Corp., version 25) was used for all the above-mentioned evaluations. A *p* < 0.05 was considered to indicate statistical significance.

## 3. Results

### 3.1. Patients’ Characteristics

Sixty-five patients were enrolled in the study (Figure 1). Patient characteristics are detailed in Table 1.

Tumor site reconstruction required a pedicle flap in 24 cases (19 pectoralis major myocutaneous flaps, 5 supraclavicular artery island flap), a free flap in 9 (7 radial forearm free flap, one latissimus dorsi myocutaneous flap and one anterolateral thigh flap) and a double flap in 4 (3 fibula free flaps with pectoralis major myocutaneous flaps, one radial forearm free flap with Mustardé rotational cheek flap). In 28 cases the tumor site was managed by primary closure. A montgomery salivary bypass tube was systematically used in total laryngectomy patients only in salvage settings.

### 3.2. Thirty-Day Major Postoperative Complications

The 30-day major postoperative complication rate was 35% (23/65). These complications included thirteen pharingo-cutaneous fistula (PFC), four respiratory failures (for acute pneumonias, pleural effusion, pulmonary embolism), three partial flap failures, two septic shocks, two cardiac arrests, one chyle leak and two oro-cutaneous fistula (OCF). For statistical analysis, patients who reported multiple complications were recorded with the most serious one. PCF (*n* = 13) were managed by conservative treatment (compressive dressings, long-lasting Montgomery salivary bypass tube) in four cases, by secondary pectoralis major myocutaneous flap (PMMCF) and placement of Montgomery salivary bypass tube in two cases, by secondary supraclavicular artery island flap (SCAIF) in three cases, by surgical debridement of flap necrosis and primary closure with Montgomery salivary bypass tube placement in four cases. Partial flap failures (*n* = 3) were managed by surgical debridement of flap necrosis in two cases and by secondary PMMCF with Montgomery salivary bypass tube in one case. The chyle leak was managed by primary duct closure and concurrent onlay PMMF. OCF (*n* = 2) were managed by conservative treatment (compressive dressings, primary suture). There were four deaths in the thirty-day postoperative period as a consequence of septic or cardiac conditions. Median hospital stay was 15 days (IQR 12). It was significantly longer in complicated patients (29 [IQR 21] days vs. 14 [IQR 4] days; *p* < 0.001).

### 3.3. Sarcopenia and 30-Day Major Postoperative Complications

**Figure 4:**
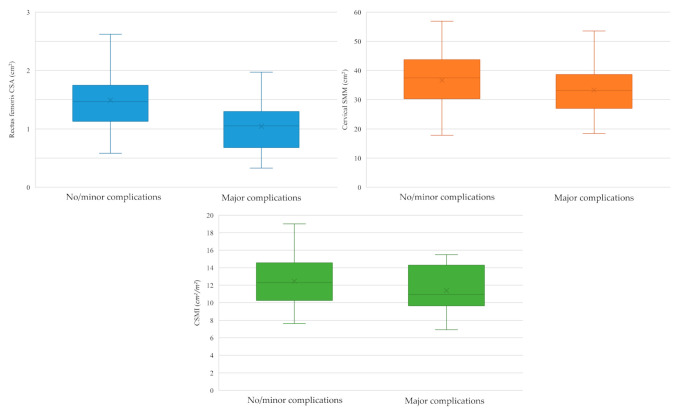
Box-plots depicting ultrasonographically-defined rectus femoris cross sectional area (upper left, cm^2^), CT/MRI-defined cervical skeletal muscle mass (upper right, cm^2^) and CT/MRI-defind cervical skeletal muscle index (lower, cm^2^/m^2^) as stratified by the occurrence of 30-day major postoperative complications; CSA: cross sectional area; SMM: skeletal muscle mass; CSMI: cervical skeletal muscle index.

We singularly analyzed US-defined rectus femoris CSA and CT/MRI-defined cervical SMM and CSMI as surrogate markers of sarcopenia, assessing their association with the occurrence of major postoperative complications. Box-plots are shown in Figure 4. Considering US-defined SMM, in patients with no/minor complications rectus femoris CSA was 1.49 ± 0.49 cm^2^, while in patients who experienced major complications 1.04 ± 0.44 cm^2^ (*p* = 0.001). As far as CT/MRI-defined cervical SMM was concerned, it was higher in patients with no/minor complications (36.66 ± 9.29 cm^2^) than in those who suffered from major complications (33.25 ± 8.51 cm^2^), though not in a statistically significant manner (*p* = 0.151). The same trend was noted for CSMI (12.46 ± 2.77 cm^2^/m^2^ in patients with no/minor complications vs. 11.38 ± 2.48 cm^2^/m^2^ in patients with major complications; *p* = 0.124) (Figure 4).

**Figure 5:**
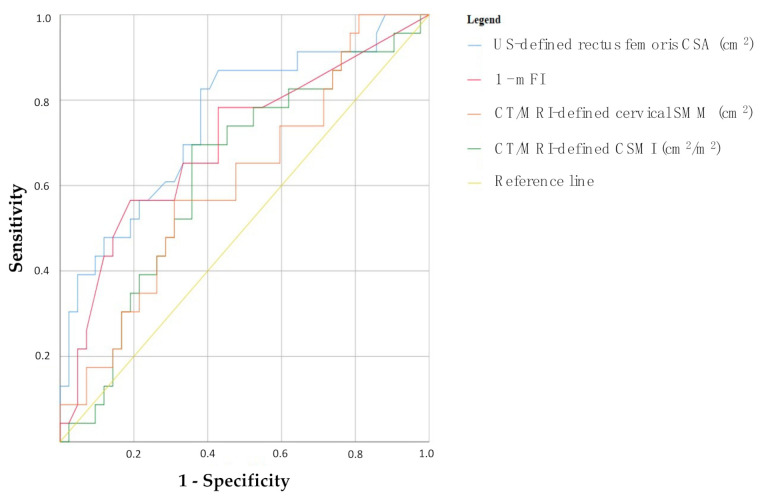
Receiver-operating characteristic curves of ultrasonographically-defined rectus femoris cross sectional area, CT/MRI-defined cervical skeletal muscle mass, CT/MRI-defined cervical skeletal muscle index and modified frailty index in terms of best prediction of 30-day major postoperative complications; US: ultrasound; CT: computed tomography; MRI: magnetic resonance imaging; SMM: skeletal muscle mass; CSMI: cervical skeletal muscle index.

ROC curves were designed to evaluate the accuracy of these three methods (together with mFI, the only continuous variable used to clinically define frailty) in terms of best prediction of 30-day major postoperative complications (Figure 5). All the considered measurements of sarcopenia and frailty showed a substantial degree of prediction of the variable of outcome, as expressed by their area under the curve (AUC), though only US-defined rectus femoris CSA and mFI reached statistical significance:

- US-defined rectus femoris CSA: AUC = 0.754, *p* = 0.001 (95% CI 0.63–0.88);
- 1-mFI: AUC = 0.699, *p* = 0.008 (95% CI 0.56–0.84);
- CT/MRI-defined cervical SMM: AUC = 0.629, *p* = 0.086 (95% CI 0.49–0.77);
- CT/MRI-defined CSMI: AUC = 0.612, *p* = 0.138 (95% CI 0.47–0.75).

According to the mentioned ROC curves of Figure 5, Youden test was used to define the cut-offs for the definition of low SMM, as surrogate marker of sarcopenia:

- US-defined rectus femoris CSA: 1.32 cm^2^ (sensitivity 82%, specificity 62%, VPP 55%, VPN 87%; prevalence of low SMM: 54%, 35/65);
- CT/MRI-defined cervical SMM: 34.91 cm^2^ (sensitivity 70%, specificity 64%, VPP 52%, VPN 79%; prevalence of low SMM: 48%, 31/65);
- CT/MRI-defined CSMI: 11.25 cm^2^/m^2^ (sensitivity 57%, specificity 69%, VPP 50%, VPN 74%; prevalence of low SMM: 40%, 26/65).

### 3.4. Clinical Predictors of 30-Day Major Postoperative Complications

The occurrence of major postoperative complications with dichotomized clinical variables was analyzed through association studies, as illustrated in Table 2.

At univariate analysis, the following parameters were found to be statistically significant predictors of 30-day major postoperative complications: previous radiotherapy (*p* = 0.038, OR 4.16, 95% CI 1.10–16.20) ASA score (*p* = 0.061, OR 2.60, 95% CI 0.91–7.39), mFI (*p* = 0.013, OR 3.75, 95% CI 1.28–10.95), US-defined rectus femoris CSA (*p* = 0.001, OR 7.72, 95% CI 2.22–26.81), CT/MRI-defined cervical SMM (*p* = 0.009, OR 4.11, 95% CI 1.38–12.23) and CSMI (*p* = 0.044, OR 2.90, 95% CI 1.01–8.31).

Considering a model including previous radiotherapy, ASA score and mFI, only US-defined rectus femoris CSA was confirmed as independent predictor of 30-day major postoperative complications at multivariate analysis (*p* = 0.004, OR 7.07, 95% CI 1.87–26.67). With a similar model including previous radiotherapy, ASA score and mFI, also CT/MRI-defined cervical SMM was proved as the only significant predictor of 30-day major postoperative complications (*p* = 0.005, OR 6.74, 95% CI 1.80–25.23). The same was evident for CT/MRI-defined CSMI with a model including previous radiotherapy, ASA score and mFI (*p* = 0.025, OR 4.02, 95% CI 1.19–13.56) (Table 3).

## 4. Discussion

Sarcopenia and its relationship to surgical morbidity is a relatively recent concept, which has been pioneered in general surgery with a growing body of literature suggesting its role as a major risk factor for postoperative complications in esophageal [11], colorectal [12], gastric [13], pancreatic [14], hepatocellular [15] and peritoneal cancer surgery [22]. HNSCC patients are among the individuals at highest risk for the development of malnutrition and sarcopenia, especially those with locally-advanced diseases. Indeed, LA-HNSCC and its treatment modalities can seriously compromise patients’ nutritional status: approximately 35% to 60% of all patients with head and neck cancer are malnourished at the time of their diagnosis [5,23], with each of the potential treatment strategy encompassing eventual side effects that may synergistically contribute to the disease-related malnutrition [6,24]. In fact, severity of malnutrition correlates with the tumor burden, affecting the chances of survival [7]. In a stage III-IV head and neck cancer cohort, Mick et al. proved that the strongest independent predictor of survival was indeed pre-treatment weight loss [25]. Nevertheless, the attention that is paid to SMM depletion and its detrimental consequences on HNSCC treatment is still limited. This could be related to the fact that the presence of sarcopenia has been traditionally assessed through cross-sectional imaging (CT/MRI) performed at the level of L3 [11,12,13,14,15,22], an analysis which is very convenient for general surgeons, but which may be troublesome in the field of head and neck because of the lack of abdomen imaging in the regular preoperative workup of such patients. Despite this, some works have been published even for HNSCC patients: for instance, Achim et al. used CT-defined L3 SMM in a 70-case cohort of total laryngectomy patients to prove that sarcopenia increased chances of developing PCF (OR 1.32, 95% CI 1.13–1.53) and wound complications per se (OR 7.54, 95% CI 1.56–36.4), being the only significant predictor for all complications on univariate and multivariate analysis [26]. Also, Jung et al. in a 190-case cohort of head and neck cancer patients aged ≥ 65 years submitted to curative surgery found that CT-defined L3 SMM revealed a subset of sarcopenic subjects with a 3.2-fold increase in the early complication rate and a 4.5-fold increase in mortality [27].

The recent findings of Swartz et al. have allowed us to overcome the aforementioned limitation; they proved that the evaluation of SMM and sarcopenia was also feasible using head and neck imaging, with C3 as an alternative reference point to L3 [16]. Consequently, Bozkurt et al. used CT-defined C3 SMM to stratify a cohort of total laryngectomy patients detecting a significant difference in neck muscle CSA between complicated (770.53 mm^2^/m^2^, 95% CI 691.16–849.90) and uncomplicated patients (875.11 mm^2^/m^2^, 95% CI 815.25–934.97; *p* = 0.043), without confounding differences in inter-individual variables (T stage, BMI, albumin levels, age, smoking) [28]. The same concept was applied by Bril et al.; they took advantage of CT/MRI-defined C3 SMM in a 235-case cohort of total laryngectomy patients to define a sarcopenic subset (46.4%) that experienced higher chances of developing PCF than their non-sarcopenic counterparts (34.9% vs. 20.6%; *p* = 0.02), also with longer hospitalizations (median, 17 vs. 14 days; *p* < 0.001) [29].

Estimating SMM through cross-sectional imaging has unquestionable advantages; firstly, the possibility to perform retrospective analysis on historical cohorts without requiring further diagnostics. Nevertheless, this assessment relies on a dedicated, expensive software for CT/MRI images analysis (e.g., SliceOmatic, Volumetool Research, Philips Intellispace Portal), not everywhere available. Moreover, the frequent occurrence of dental artefacts at C3 level may impair the proper segmentation of cervical SMM. Finally, the impossibility of standardizing so-called “exam-to-surgery time” and to perform ongoing reassessments during eventual pre-habilitation strategies are limitations that should always be considered. As a consequence, looking for alternative techniques that could be introduced in everyday clinical practice to reliably assess SMM in head and neck cancer patients become of paramount importance. In 2016, Mueller et al. first proposed US-defined sex-adjusted CSA of rectus femoris muscle as a tool to identify sarcopenic patients (43.1% of their subset) and predict adverse discharge dispositions/in-hospital mortality (OR 7.49, 95% CI: 1.47–38.24; *p* = 0.015) and longer hospital stay in a cohort of surgical intensive care unit patients, just as effectively as the Frailty Index did [19]. Consequently, in a previous work we tried doing the same in a head and neck cancer surgical cohort, proving that US-defined CSA of rectus femoris muscle was significantly lower in complicated cases (0.95 ± 0.48 cm^2^) in comparison to uncomplicated ones (1.41 ± 0.49 cm^2^; *p* = 0.003). We also detected a significant correlation between CSA and preoperative albumin levels (Spearman coefficient 0.338; *p* = 0.020), which is still one of the most effective serum markers of cancer-related systemic inflammation and cachexia according to the European Society for Clinical Nutrition and Metabolism (ESPEN) guidelines [30]. This latter finding highlighted the potential of US-defined CSA of rectus femoris muscle as a surrogate marker of frailty, leading to the identification of a sarcopenic subset of patients characterized by a higher rate of postoperative complications (77.8% vs. 27.6%, OR 9.19, 95% CI 2.32–36.43; *p* = 0.001), major complications (50.0% vs. 17.2%, OR 4.80, 95% CI 1.26–18.24; *p* = 0.024) and deaths (22.2% vs. 0.0%, OR 1.29, 95% CI 1.01–1.65; *p* = 0.017) [17]. The present study confirmed that SMM depletion was related to an augmented incidence of 30-day major postoperative complications in LA-HNSCC patients, regardless of how sarcopenia was defined. Indeed, at univariate analysis US-defined rectus femoris CSA, CT/MRI-defined cervical SMM and CSMI were all proven to be statistically significant predictors of major complications, as well as previous radiotherapy, ASA score and mFI. Likewise, at multivariate analysis SMM depletion was confirmed as significant predictor of major complications in three different models including previous radiotherapy, ASA score and mFI, either defined ultrasonographically (US-defined rectus femoris CSA: OR 7.07, 95% CI 1.87–26.67; *p* = 0.004) or by neck cross-sectional imaging (CT/MRI-defined cervical SMM: OR 6.74, 95% CI 1.80–25.23; *p* = 0.005; CT/MRI-defined CSMI: OR 4.02, 95% CI 1.19–13.56; *p* = 0.025).

To the best of our knowledge, this is the first study that puts together US of rectus femoris muscle and neck CT/MRI on the same cohort of HNSCC patients to estimate in parallel SMM and define a sarcopenic subset. Though this analysis was not meant to demonstrate the superiority of one measurement over the other in terms of sample size and design of the study, US-defined sarcopenia may provide several theoretical advantages over the CT/MRI-defined one in the preoperative workup of head and neck cancer patients thanks to its bedside feasibility (during out-patient consultation or hospital admittance), low cost, quick execution (about 5 min), no further biological risk and standardizable “exam-to-surgery time”. Finally, since sarcopenia is a potentially modifiable risk factor, US of rectus femoris muscle could offer a repeatable parameter to follow in the perioperative settings to verify the effects of any pre-habilitation strategy. This latter would be impossible with neck cross-sectional imaging, due to unacceptable additional biological risk. Despite these remarks, a further analysis powered for superiority assessment will be needed to eventually prove the better sensitivity of ultrasonographically defined over cross-sectional sarcopenia.

This work presents some limitations. Obviously, the narrow sample size (*n* = 65) and number of outcome events (i.e., 30-day major postoperative complications) limited the independent variables that could be included in the multivariate analysis model and prevented to perform subset analysis on specific complications and surgical procedures. Moreover, some kind of selection bias was introduced due to the need for both diagnostic US and neck cross-sectional imaging in the preoperatory workup of patients enrolled for the study. Finally, a comprehensive nutritional assessment (e.g., Patient-Generated Subjective Global Assessment) and a concurrent evaluation of muscle strength (e.g., handgrip strength, knee flexion/extension testing) and/or physical performance (e.g., gait speed, the Short Physical Performance Battery) were lacking.

## 5. Conclusions

To date, SMM depletion and sarcopenia represent effective predictors of postoperative major complications in many oncological scenarios, even in the field of head and neck. As previously demonstrated, a huge of portion of LA-HNSCC patients (stage III–IV) develop sarcopenia during the natural history of their disease, for both cancer-related and treatment-related reasons. In this subset of patients, SMM depletion either defined through US (as CSA of rectus femoris muscle) or neck cross-sectional imaging (as CSA at C3 level or CSMI) was proved to significantly predict 30-day major postoperative complications better than any other clinical of functional parameter, also providing a fundamental tool for ongoing reassessment of patients with regards to any pre-habilitation strategy aimed at reducing surgical morbidity. Further prospective, multicentric studies will be needed to define shared cut-offs to apply to the definition of sarcopenia and to properly assess the eventual differences in terms of sensitivity between such techniques.

## Figures and Tables

**Table 1:** Patient characteristics. PT: pull-through; TM: trans-mandibular; OPHL: open partial horizontal laryngectomy; TL: total laryngectomy; PP: partial pharyngectomy.

Parameters	Values (%)	Means ± SD
**Age**		65.9 ± 12.2 years
**<70 years** **≥70 years**	38 (58) 27 (42)	-
**Gender**		
**Male** **Female**	53 (81) 12 (19)	-
**Tumor subsite**		
**Oral cavity/oropharynx** **Larynx/hypopharynx** **Parotid**	18 (28) 45 (69) 2 (3)	-
**Tumor origin**		
**Primary** **Persistence/relapse**	46 (71) 19 (29)	-
**Surgical procedure**		
**PT/TM resection** **OPHL** **TL ± PP** **Extended parotidectomy**	18 (28) 3 (5) 42 (64) 2 (3)	-
**Previous radiotherapy**		
**No** **Yes**	54 (83) 11 (17)	-
**Body mass index**		25.3 ± 3.6 Kg/m^2^
**<18.5 Kg/m^2^** **18.5–24.9 Kg/m^2^** **25–29.9 Kg/m^2^** **≥30.0 Kg/m^2^**	2 (3) 29 (45) 29 (45) 5 (7)	-
**Smoking habit**		-
**<20 pack-year** **≥20 pack-year**	21 (32) 44 (68)	-
**Diabetes mellitus**		
**No** **Yes**	53 (81) 12 (19)	-
**Cholinesterase (ChE)**		7.4 ± 1.87 kU/L
**Creatine phosphokinase (CPK)**		69.25 ± 33.65 U/L
**Hemoglobin (Hb)**		13.41 ± 1.68 g/dL
**Weight loss within last 3 months**		
**<10%** **≥10%**	60 (92) 5 (8)	-
**Surgical time**		373.3 ± 127.8 min
**<300 min** **≥300 min**	21 (32) 44 (68)	-
**Albumin (Alb)**		34.64 ± 6.53 g/L
**>35.00 g/L** **≤35.00 g/L**	35 (54) 30 (46)	-
**pTNM stage**		
**III** **IVa** **IVb**	20 (31) 28 (43) 17 (26)	-
**pN class**		
**pN0** **pN+**	34 (52) 31 (48)	-
**Free flap reconstruction**		
**No** **Yes**	52 (80) 13 (20)	-
**ASA score**		
**I** **II** **III** **IV**	1 (2) 37 (57) 25 (38) 2 (3)	-
**Charlson comorbidity index (CCI)**		
**<5** **≥5**	25 (39) 40 (61)	-
**Modified frailty index (mFI)**		
**0–0.05** **0.05–0.10** **0.10–0.15** **0.15–0.20** **>0.20**	19 (29) 17 (26) 14 (22) 6 (9) 9 (14)	-

**Table 2:** Thirty-day major postoperative complications and dichotomized clinical predictors, univariate analysis; * Fisher’s exact test or Chi-squared test, as appropriate. BMI: body mass index; ASA: American Society of Anesthesiologists; CSA: cross-sectional area; SMM: skeletal muscle mass; CSMI: cervical skeletal muscle index.

Parameters	30-Day Major Complications		OR (95% CI)	p *
	No (%)	Yes (%)		
**Age**				
**<70 years** **≥70 years**	24 (63) 18 (67)	14 (37) 9 (33)	1 [reference] 0.86 (0.30–2.416)	0.771
**Previous radiotherapy**				
**No** **Yes**	38 (70) 4 (36)	16 (30) 7 (64)	1 [reference] 4.16 (1.10–16.20)	0.038
**BMI**				
**≥18.5 Kg/m^2^** **<18.5 Kg/m^2^**	41 (65) 1 (50)	22 (35) 1 (50)	1 [reference] 1.87 (0.11–31.26)	0.586
**Smoking habit**				
**<20 pack-year** **≥20 pack-year**	13 (62) 29 (66)	8 (38) 15 (34)	1 [reference] 0.84 (0.29–2.47)	0.752
**Diabetes mellitus**				
**No** **Yes**	37 (70) 5 (42)	16 (30) 7 (58)	1 [reference] 3.24 (0.89–11.75)	0.068
**Weight loss within last 3 months**				
**<10%** **≥10%**	39 (65) 3 (60)	21 (35) 2 (40)	1 [reference] 1.24 (0.19–8.00)	0.585
**Surgical time**				
**<300 min** **≥300 min**	16 (76) 26 (59)	5 (24) 18 (41)	1 [reference] 2.21 (0.69–7.14)	0.178
**Albumin (Alb)**				
**>35.00 g/L** **≤35.00 g/L**	24 (69) 18 (60)	11 (31) 12 (40)	1 [reference] 1.45 (0.52–4.04)	0.471
**pTNM stage**				
**III** **IVa-IVb**	13 (65) 29 (64)	7 (35) 16 (36)	1 [reference] 1.03 (0.34–3.09)	0.966
**Free flap**				
**No** **Yes**	34 (65) 8 (61)	18 (35) 5 (39)	1 [reference] 1.18 (0.33–4.14)	0.518
**ASA score**				
**I–II** **III–IV**	28 (74) 14 (52)	10 (26) 13 (48)	1 [reference] 2.60 (0.91–7.39)	0.061
**Charlson comorbidity index (CCI)**				
**<5** **≥5**	18 (72) 24 (60)	7 (28) 16 (40)	1 [reference] 1.71 (0.58–5.04)	0.325
**Modified frailty index (mFI)**				
**≤0.10** **>0.10**	28 (78) 14 (48)	8 (22) 15 (52)	1 [reference] 3.75 (1.28–10.95)	0.013
**Ultrasonographically-defined** **rectus femoris CSA (cm^2^)**				
**>1.32 cm^2^** **≤1.32 cm^2^**	26 (87) 16 (46)	4 (13) 19 (54)	1 [reference] 7.72 (2.22–26.81)	0.001
**CT/MRI-defined cervical SMM (cm^2^)**				
**>34.91 cm^2^** **≤34.91 cm^2^**	27 (79) 15 (48)	7 (21) 16 (52)	1 [reference] 4.11 (1.38–12.23)	0.009
**CT/MRI-defined CSMI (cm^2^/m^2^)**				
**>11.25 cm^2^/m^2^** **≤11.25 cm^2^** **/m^2^**	29 (74) 13 (50)	10 (26) 13 (50)	1 [reference] 2.90 (1.01–8.31)	0.044

**Table 3:** Thirty-day major postoperative complications and dichotomized clinical predictors, multivariate analysis (multiple logistic regression); ASA: American Society of Anesthesiologists; CSA: cross-sectional area; SMM: skeletal muscle mass; CSMI: cervical skeletal muscle index.

Parameters	OR (95% CI)	*p*	OR (95% CI)	*p*	OR (95% CI)	*p*
**Previous radiotherapy**						
**No** **Yes**	1 [reference] 3.22 (0.56–18.49)	0.190	1 [reference] 2.35 (0.43–12.93)	0.327	1 [reference] 2.32 (0.46–11.76)	0.308
**ASA score**						
**I–II** **III–IV**	1 [reference] 1.42 (0.38–5.26)	0.599	1 [reference] 1.53 (0.41–5.66)	0.525	1 [reference] 1.35 (0.38–4.82)	0.648
**Modified frailty index (mFI)**						
**≤0.10** **>0.10**	1 [reference] 1.54 (0.36–6.63)	0.560	1 [reference] 3.75 (0.79–17.77)	0.096	1 [reference] 3.21 (0.73–14.19)	0.124
**US-defined rectus femoris CSA (cm^2^)**						
**>1.32** **≤1.32**	1 [reference] 7.07 (1.87–26.67)	0.004	-	-	-	-
**CT/MRI-defined cervical SMM (cm^2^)**						
**>34.91** **≤34.91**	-	-	1 [reference] 6.74 (1.80–25.23)	0.005	-	-
**CT/MRI-defined CSMI (cm^2^/m^2^)**						
**>11.25** **≤11.25**	-	-	-	-	1 [reference] 4.02 (1.19–13.56)	0.025

## Data Availability

The data presented in this study are available on request from the corresponding author.
